# Bioavailability, Human Metabolism, and Dietary Interventions of Glucosinolates and Isothiocyanates: Critical Insights and Future Perspectives

**DOI:** 10.3390/foods14162876

**Published:** 2025-08-19

**Authors:** Federica Narra, Giulia Galgani, Cassidy Bo Harris, Diego A. Moreno, Vanesa Núñez-Gómez

**Affiliations:** 1 Department of Agriculture, Food and Environment, University of Pisa, Via del Borghetto 80, 56124 Pisa, Italy; federica.narra@phd.unipi.it; 2 Department of Pharmacy, University of Pisa, Via Bonanno 6, 56126 Pisa, Italy; giulia.galgani@phd.unipi.it; 3 Research Group Phytochemistry and Healthy Food Lab (LabFAS), CSIC, CEBAS, Campus Universitario de Espinardo 25, 30100 Murcia, Spain; cassidybo.harris@um.es (C.B.H.); vngomez@cebas.csic.es (V.N.-G.)

**Keywords:** glucosinolate, sulforaphane, isothiocyanate, bioavailability, microbiota, epithionitriles, indole-3-carbinol, clinical trials, functional foods, personalised nutrition

## Abstract

Glucosinolates (GSLs) and their breakdown products, isothiocyanates (ITCs), are bioactive compounds with anti-inflammatory, antioxidant, and anticancer properties, mediated through key pathways such as Nrf2, NF-κB, and epigenetic regulation. However, their limited and variable bioavailability remains a key challenge. This review summarises the current clinical evidence on GSLs and ITCs, with a focus on their health effects and metabolic fate in humans. Recent findings on enzymatic and microbial metabolism are discussed, along with results from interventions involving whole vegetables, sprouts, and extracts. Although promising effects on blood pressure, lipid profiles, and glycaemic control have been observed, clinical studies are often limited by small sample sizes, study heterogeneity, and high inter-individual variability, particularly related to gut microbiota and host metabolic phenotype. Challenges like inconsistent biomarkers, formulation variability, and tolerability issues complicate data interpretation. To realise their full potential, larger, standardised, microbiome-informed trials with validated biomarkers and optimised delivery are needed to clarify host–compound–microbiome interactions and support evidence-based disease prevention strategies.

## 1. Introduction

Glucosinolates (GSLs) are a diverse group of β-thioglucoside N-hydroxysulphate secondary metabolites predominantly found in cruciferous vegetables (*Brassicaceae* family), such as kale, broccoli, cauliflower, radishes, and mustards. These compounds are produced by plants in the order Brassicales and in some species of Malpighiales, which is an example of parallel evolution [1]. Beyond their role in plant defence against pathogens, GSLs have drawn considerable interest for their potential health-promoting properties [2].

The structural diversity of GSLs arises from their amino acid precursors, allowing their classification into three main types: aliphatic (from methionine), aromatic (from phenylalanine), and indole GSLs (from tryptophan) [3,4]. Despite these differences, all GSLs share a common core structure: a thiohydroximate group with a sulphate ester at the oxygen atom and a glucose moiety linked to the sulphur atom [3,4]. The biosynthesis of GSLs occurs in three major stages. First, a chain elongation of the precursor amino acid takes place (mainly affecting non-tryptophan-derived GSLs), which may repeat up to six cycles. Second, the core GSL structure is assembled. Finally, side-chain modifications—such as hydroxylation, methoxylation, oxidation, desaturation, or benzoylation—confer further structural and functional diversity [3,5].

The primary and most abundant GSL found in plants is glucoraphanin (GR), which is derived from methionine. Other commonly occurring GSLs include sinigrin, glucobrassicin, gluconasturtiin, and glucotropaeolin, as detailed in Table 1 [6,7]. GSLs are hydrolysed by myrosinase (thioglucosidase, CAS No. 9025-38-1), leading to the formation of isothiocyanates (ITCs) [8,9] The main ITC present in these foods is sulforaphane (SFN), which is derived from GR. SFN is well known for activating the nuclear factor erythroid 2-related factor 2 (Nrf2) pathway, leading to increased cellular glutathione (GSH) levels and the induction of antioxidant enzymes [10,11]. This activation contributes to improved regulation of inflammation, hepatic lipogenesis, and gluconeogenesis [12]. Additionally, SFN has been shown to inhibit histone deacetylase (HDAC) activity in various types of cancer, highlighting its potential role in cancer prevention and therapy [13,14]. Other molecular targets of ITCs include nuclear factor kappa B (NF-κB), mitogen-activated protein kinase (MAPK), and Kelch Like ECH Associated Protein 1 (Keap1), illustrating their pleiotropic effects on cell signalling, apoptosis, and redox homeostasis [13,14].

Other abundant GSLs are shown in Table 1, along with their respective ITCs. Phenyl-ITC (PEITC) is the metabolite formed from gluconasturtiin. It plays a critical role in modulating inflammatory cytokines, such as interleukin (IL-1β, IL-6) and tumour necrosis factor (TNF-α), leading to a reduced inflammatory response to a foreign antigen [16]. Interestingly, PEITC has also demonstrated potential in modulating epigenetic mechanisms, including the inhibition of DNA methyltransferases (DNMTs), adding another layer to its chemopreventive potential [16]. Recent literature further supports these findings, highlighting PEITC’s dual role in inflammation suppression and epigenetic regulation, as well as its broader therapeutic potential in cancer and chronic inflammatory diseases [16].

Another relevant aspect of ITCs is their structure, as well as the GSLs, as it conditions their volatility and stability and has certain effects on taste and flavour [15]. The side chain of the GSL plays a crucial role in determining the type and stability of the resulting ITCs. For example, the predominant GSL in white mustard (*Sinapis alba*), glucosinalbin, produces an unstable ITC with a half-life of only a few hours. In contrast, black mustard (*Brassica nigra*) contains sinigrin as its main GSL, which yields allyl-ITC (AITC), a compound with significantly greater stability [3]. These chemical properties not only affect the sensory profile of the vegetables but also their functional properties, bioavailability, and therapeutic potential [3].

In addition to these physicochemical characteristics, the biological activity of ITCs is strongly influenced by their chemical structure. Different GSL-derived ITCs act through distinct molecular pathways, highlighting the need to consider their structural diversity when evaluating health effects. For instance, certain ITCs have been shown to modulate tumour suppressor pathways, such as PTEN via inhibition of MYC-WWP1, as reported by Lee et al. [17]. Furthermore, specific gut microbes have the capacity to selectively metabolise GSLs into active ITCs, reinforcing the relevance of both compound structure and host microbiota in shaping bioactivity [18].

GSLs are widely present in cruciferous vegetables, but recent research has identified additional dietary sources and metabolites. Notably, Brassica microgreens—edible seedlings with developed cotyledons, such as broccoli and radish sprouts—have gained attention for their rich phytochemical profile, including high levels of GSLs, phenolic compounds, carotenoids, and vitamins [19,20]. Other non-Brassicaceae sources, such as capers (*Capparis spinosa*), moringa (*Moringa oleifera*), and papaya seeds (*Carica papaya* L.), also contain GSLs. *Moringa oleifera*, [21] in particular, has emerged as a distinctive source of isothiocyanates (ITCs), notably 4-(α-L-rhamnopyranosyloxy)-benzyl isothiocyanate (moringin or MIC-1), which includes an uncommon rhamnose sugar moiety. This structural feature is rare among ITCs and plays a key role in enhancing the compound’s solubility, metabolic stability, and bioavailability. These properties may contribute to the potent anti-inflammatory and chemoprotective effects observed in preclinical studies, reinforcing its potential value as a non-conventional dietary source of bioactive ITCs [22,23]. GSLs and ITCs are also found in processed products like rapeseed and canola oils, mustard sauce, and sauerkraut [24]. A recent systematic review of human studies conducted by Costa-Pérez et al. [25] has reinforced the growing interest in GSLs and their bioactive derivatives due to their metabolic relevance in human health. Evidence suggests that dietary intake of cruciferous vegetables is associated with measurable changes in clinical and biochemical markers, including reductions in oxidative stress, inflammation, and improvements in lipid and glucose metabolism. Moreover, the bioavailability and physiological impact of ITCs appear to be strongly influenced by several variables, such as the method of vegetable preparation (e.g., raw vs. cooked), individual GMB composition, and the presence or absence of active plant myrosinase. Notably, interindividual variability in ITC metabolism has been linked to differences in enzymatic hydrolysis efficiency and microbiota-driven activation of GSLs. Despite the promising results, the review emphasises the need for further studies and reviews to better define the therapeutic potential and safety of GSL-derived compounds in humans.

In this context, an updated and integrative review is essential to summarise recent advances in the understanding of GSL metabolism, structure-activity relationships, and their multifaceted biological effects. Given the rapidly evolving landscape of research—including new insights into molecular mechanisms, GMB interactions, and epigenetic modulation—a comprehensive synthesis of current evidence is crucial for guiding future research and informing dietary recommendations and therapeutic strategies.

Based on this background, this review aims to provide an updated and comprehensive overview of the clinical studies carried out based on GSLs and their derivatives, with particular emphasis on their role in human health. This work seeks to integrate recent findings from biochemical, nutritional, and clinical research in order to clarify the underlying mechanisms. It also aims to serve as a comprehensive and accessible reference for current developments in this growing but still underexplored field linking food science and human health.

## 2. Methodology

A literature search was conducted in three major scientific databases: Scopus^®^ (Elsevier, Amsterdam, Netherlands), PubMed^®^ (National Library of Medicine, Bethesda, MD, USA), and Web of Science (Clarivate Analytics, London, UK), in April 2025. The objective was to identify relevant publications focused on human studies involving the intake of Brassicaceae foods or related formulations—vegetables and derivatives, sprouts, and enriched foods and extracts—as dietary sources of GSLs and ITCs, with an emphasis on their metabolism, bioavailability, and potential physiological effects. In addition to clinical trials assessing ITC metabolites in biological fluids (e.g., plasma or urine), studies investigating the interaction of GSLs and ITCs with the gut microbiota were also included, given the increasing evidence supporting the microbiota’s role in modulating the metabolism and bioactivity of these compounds. Studies were selected based on the following inclusion criteria: (i) human interventions using Brassicaceae foods or derived products; (ii) availability of data on vital signs or GSL or ITC metabolites in biofluids; (iii) studies evaluating outcomes related to gut microbiota composition or function; and (iv) studies conducted in healthy individuals or populations with specific health conditions. The search strategy combined the terms (“glucosinolate” OR “isothiocyanate” OR “indole”) AND (“bioavailability” OR “metabolism” OR “pharmacokinetics”) AND (human) AND (“urine” OR “plasma” OR “gut microbiota”) and covered the period from 2020 to 2025. Exclusion criteria included animal or in vitro studies, reviews, trial protocols without results, and studies lacking relevant metabolite or microbiota data. Article selection was performed independently by three researchers, and disagreements were resolved through discussion with a fourth author. After title and abstract screening and full-text evaluation, a total of 24 studies were included, out of the 63 initially identified through the database search. The specific information regarding the dietary source is depicted in tables; type and dose of compounds; type of biological sample; subject characteristics; study record identifier (NCT (number in ClinicalTrials.gov Database) number); concentration of metabolites excreted; main findings; analytical technique used; and reference identification number.

## 3. Metabolism, Bioavailability, and Bioaccessibility of GSLs and ITCs

### 3.1. Metabolism and Myrosinase Activity

Understanding the mechanisms that lead to the production and absorption of GSL metabolites is crucial for their application as health promoters [26,27,28]. The first step of GSL metabolism is hydrolysis, catalysed mostly by plant myrosinase, an enzyme belonging to the family of β-thioglucosidase, but also by gut myrosinase-like enzymes. This enzyme, stored in vegetal cells in separate compartments from GSLs, is able to hydrolyse the glycosidic bond to obtain glucose and several different breakdown products, such as ITCs, nitriles, epithionitriles, and other minor degradation products, such as thiocyanates and, for indolyl GSLs, indolic-derived sub-products [29]. In healthy plant conditions, the activity of myrosinase is absent; however, when the cell loses its structure, either due to toxic or physical damage, myrosinase and GSLs are released, permitting a rapid hydrolysis of the compounds toward their degradation products. Despite the main interest of researchers being focused on hydrolysis products, which show several properties useful in different fields, mostly pharmaceutical and agricultural purposes, these compounds show high volatility and less stability in water compared to parental GSLs. Therefore, a thorough understanding of myrosinase properties is crucial for maximising the yield of bioactive compounds derived from GSL. As myrosinase needs to get in touch with GSLs to exert its effects, processing the samples before their use plays a major role in their properties. Myrosinase can be inactivated by high temperatures and pressures, both alone and in combination [30], thus leading to further investigation on cooking procedures of the materials to preserve their properties. Oloyede et al. [31] evaluated the effects of steaming, microwaving, and stir-frying on myrosinase stability in *Brassica oleracea*. They demonstrated that stir-frying showed the best myrosinase activation, resulting in the lowest GSL content, while steaming leads to the highest GSL content and myrosinase inactivation.

**Figure 1:**
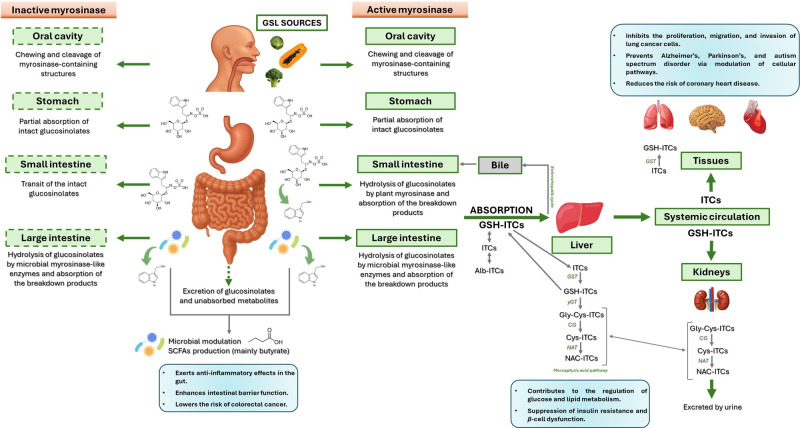
Overview of the metabolism of glucosinolates and their breakdown products from dietary sources, including processes from chewing, absorption, and hydrolysis in the gut, to microbial hydrolysis and metabolism under conditions of active and inactive myrosinase, as well as their subsequent beneficial effects on target organs, adapted from Barba et al. [32] and Esteve et al. [6] GSLs: glucosinolates; ITCs: isothiocyanates; GSH-ITCs: glutathione–isothiocyanate conjugates; Alb-ITCs: albumin–isothiocyanate conjugates; Gly-Cys-ITCs: glycine–cysteine–isothiocyanate conjugates; Cys-ITCs: cysteine–isothiocyanate conjugates; NAC-ITC: N-acetylcysteine–isothiocyanate conjugates; GST: glutathione-S-transferase; γGT: γglutamyltranspeptidase; CG: cysteinylglycinase; NAT: N-acetyltransferase.

Upon ingestion (Figure 1), mechanical disruption in the oral cavity (e.g., through mastication) leads to the disruption of plant tissue integrity, facilitating the release of endogenous myrosinase. If the vegetables are consumed raw, this enzyme may initiate the hydrolysis of GSL into bioactive compounds such as ITCs. Any intact GSLs that escape hydrolysis in the mouth may undergo limited absorption in the stomach. The remaining GSLs then transit through the gastrointestinal tract to the small intestine, where further hydrolysis may occur in the presence of residual plant myrosinase, with the resulting degradation products being available for absorption. Unhydrolysed GSLs that persist are transported to the colon, where microbial myrosinase-like enzymes activity may mediate additional hydrolysis. The resulting metabolites are subsequently absorbed and/or excreted [32].

After absorption (Figure 1), ITCs undergo phase II metabolism, primarily through conjugation with GSH, followed by their conversion to mercapturic acids and subsequent excretion [32]. One of the main challenges in harnessing the health benefits of GSLs and ITCs is improving their bioavailability. Bioavailability depends on several factors, mostly due to the chemical structure of a molecule and its interaction with the environment. Research on the use of ITCs as beneficial agents has always been slowed down by their little bioavailability, as these substances are usually insoluble, show little stability in water and at extreme pH conditions, and are thermally degraded [33,34,35].

Assessing the bioaccessible fraction (BF) of GSLs and ITCs of a given sample can be a first step to obtain bioavailable compounds. Bioaccessibility refers to the proportion of an ingested molecule that becomes available for absorption in the intestine and is represented by the aqueous content obtained at the end of the digestion procedure. It is influenced by various factors, including the food matrix, freshness, cooking methods, and molecular stability. Several studies have been conducted over the years to understand the factors that influence ITCs’ bioaccessibility, analysing their content after simulated in vitro gastrointestinal digestion, with INFOGEST as the most used protocol [36].

Several authors evaluated differences among plants on the total BF of GSLs and ITCs after in vitro digestion and the influence of cultivation or sample pre-treatment on the final content of the compounds of interest [37,38,39]. Cámara-Martos et al. [37] evaluated the differences between turnip greens and tops, composed of fructiferous stems with flower buds, harvested at maturation. Final GSL bioaccessibility, after in vitro digestion, was higher than 50% both for turnip tops and greens, with a mean of 66% for the former and 73% for the latter. High bioaccessibility of GSLs deriving from the aerial part of a plant was also demonstrated by the study conducted by Martínez-Castro et al. [38], which analysed four plants of the Brassicaceae family: Ethiopian mustard (*Brassica carinata Braun*), turnip greens and turnip tops (*Brassica rapa* var. *rapa*), rocket (*Eruca vescicaria* L.), and white mustard (*Sinapis alba* L.). They tried to understand the impact of natural or organic cultivation on GSL content in florets and leaves (3–5-month-old plants) of these plants and their bioaccessibility. The final BF of all samples varied between 60 and 78% of the initial total content, with higher results for *Brassica rapa* and *Brassica carinata* cultured in the conventional model compared to the organic model, while the contrary was seen for the other two species. On the other hand, when analysing seed content, despite an initial higher GSL content compared to the green parts of the plants, BF resulted extremely low, under 1%. However, as myrosinase was not inactivated during all the procedures, GSLs must be considered underestimated, as a slight percentage was converted to ITCs. A similar study was conducted by García-Perez et al. [39], who tried to delineate GSL bioaccessibility in microgreens of eight Brassicaceae: broccoli (*Brassica oleracea* var. *italica cv. Lvhua*), red mustard (*Brassica juncea* (L.) *Czern.*), cauliflower (*Brassica oleracea* var. *botrytis*), red cabbage (*Brassica oleracea* L. var. *capitata*), kale (*Brassica oleracea* L. var. *sabellica*), Brussels sprouts (*Brassica oleracea* var. *gemmifera*), pak choi (*Brassica rapa* subsp. *pekinensis*), and arugula (*Eruca sativa Mill.*). They evaluated the bioaccessibility after in vitro digestion and intestinal fermentation, obtained with antibiotic-free faeces of healthy donors. Results indicated that, after digestion and fermentation, GSL content was high in red cabbage, cauliflower, and pak choi, varying from a minimum of 49.3 to a maximum of 69.1 mg/g of dry weight (DW) for red cabbage, in line with higher content in raw materials. In vitro digestion was also performed by Salas-Millán et al. [40] to evaluate the potential beneficial properties of a lacto-fermented broccoli beverage, analysing SFN, polyphenol, and indole content. SFN content decreased significantly after the intestinal phase, from 2.5 mg/L in the beverage to 1.7 mg/L after 30 min of intestinal digestion and a final concentration of 1.0 mg/L at the end of the procedure, limiting the BF to 51% of the total dose. A higher reduction was evidenced for indoles, another class of GSL degradation products, whose BF has been reported to be less than 15% of their total content, limiting their availability for absorption [40].

However, the real-life bioaccessibility of Brassicaceae-derived GSLs and ITCs cannot be accurately assessed based solely on analyses of fresh material. Pre-treatment of samples, storage conditions, and cooking methods must also be taken into account. Costa-Pérez et al. [41] evaluated the bioaccessibility of organosulfur compounds derived from broccoli stalks after three different extraction methods: the first one to obtain high GSL content, the second to obtain high ITC content, and the third to obtain a more balanced content between both. After in vitro simulated gastrointestinal digestion, GSL final content was under the limit of quantification, indicating their conversion into metabolites. GR-derived ITC, SFN, was more abundant in samples whose extraction was performed to obtain high ITC content compared to high GSLs or balanced GSLs/ITCs, with a fold increase of 31.9% and 73.7%, respectively, evidencing a partial conversion of the parental molecule. In line with these results, the clinical trial conducted by Egner et al. [42], in which two broccoli beverages, one rich in SFN and the other in its parental GSL, GR, were given to fifty healthy participants, demonstrated that the final BF of SFN was higher in formulations initially richer in SFN, compared to formulations richer in GR. Thermal treatments were investigated by several authors. In a study conducted by Costa-Pérez [43], thermal drying treatment resulted in a reduction in GSL content after in vitro digestion and an increase in their breakdown products in broccoli (var. *italica*) stalk samples, suggesting the release of myrosinase during the procedure. Moreover, samples that were obtained by the freeze-drying process showed high GSL content and no signs of breakdown products, indicating that this was the best storage condition to reduce sample degradation. Similar results were obtained by Sadowska-Rociek [44], who highlighted the complete loss of GR, glucoraphenin, and gluconarstutiin, after either a conventional cooking method (boiling at 98 °C for 15 min), steaming (100 °C for 7 min), or sous-vide cooking (using vacuum packing and cooking them at 90 °C for 45–50 min), and following gastrointestinal digestion, samples were frozen, lyophilised, and ground after the cooking procedure and prior to the simulated digestion. During this phase, a reduction in GSL content was observed for the sous-vide treatment compared to the conventional cooking method. The results obtained by Vancoillie [45] showed a BF of 100% for GSLs after Brussels sprouts were heated at 95 °C in a water bath for 15 min and subjected to in vitro digestion. Although this may appear to contradict previous findings, the author emphasised that bioaccessibility refers to the percentage of a compound that can be released from the matrix, rather than the ratio of compound concentration after digestion to that before digestion, as reported in other studies. Another study, conducted by Pagliari et al. [46], evaluated the effect of pressurised liquid extraction on *Camelina sativa* (L.) *Crantz* by-products. They obtained an extract containing three GSLs (glucoarabin, glucocamelin, and homoglucocamelin) and, after in vitro simulated gastrointestinal digestion, GSL bioaccessibility, defined as the ratio between the amount after and before digestion, was 28% for glucoarabin, 26.8% for glucocamelin, and 15.7% for homoglucocamelin. Furthermore, their bioavailability reached only about 3.5%, which is indicative of significant myrosinase activity, compound degradation, and limited intestinal absorption.

### 3.2. Importance of the Gut Microbiota in GSL Metabolism

As already noted by the previous described experiments, many studies have been conducted to understand the mechanisms that allow the absorption of GSL and ITCs in the human body. Food matrices and interindividual differences—such as health status and lifestyle—are major factors influencing the variability in the metabolism of these substances. However, a less well-known yet increasingly recognised contributor to human nutrient absorption has recently drawn researchers’ attention: the GMB [47]. The GMB consists of thousands of bacterial species, each with its own metabolic activity, capable of producing a wide range of secondary metabolites within the intestine. It can replace the loss of vegetables myrosinase due to cooking or other processing methods, by exerting its myrosinase-like activity. The substantial interindividual variability in GMB composition—largely shaped by dietary patterns—can significantly influence the metabolism, bioavailability, and ultimately the effective intake of GSL and ITCs. Recent insights emphasise that specific bacterial taxa possess distinct enzymatic capabilities, such as β-thioglucosidase activity, which directly affect the hydrolysis of GSLs into biologically active ITCs. This microbial-mediated biotransformation not only affects the bioaccessibility of these compounds by modulating their release from food matrices but also their bioavailability by influencing absorption and systemic distribution. Moreover, the interaction between GSL-derived compounds and the GMB can lead to reciprocal modulation, where these metabolites alter microbial community structure and function, potentially impacting gut health and overall host metabolism [48,49].

By far, few studies have been conducted in vivo on human participants to understand the implications that GMB have on GSL metabolism (Table 2). Lee et al. [50] evaluated the effect of microgreens from red beet (*Beta vulgaris*) and red cabbage (*Brassica oleracea* var. *capitata*) on GMB in healthy middle-aged and older adults. Results showed that the intake of these substances did not affect the microbiota composition, while Bouranis et al. [48] highlighted that the conversion from GSLs to their metabolites is made mostly by bacteria from *Bifidobacterium* and *Roseburia* genera. This study was conducted analysing plasma and urine samples for SFN and faecal samples for microbiota sequencing of 55 participants in the study. They also observed that a diet rich in carbohydrates, fibres, and micronutrients may lead to a GMB composition that is more able to get GR hydrolysis [48].

**Table 2:** In vivo and in vitro studies on GMB.

Type of Study	Food Source	Aim of the Study	Experimental Plan	Main Findings	Reference
Human	Microgreens of *Beta vulgaris* and *Brassica oleracea* var *capitata*	Feasibility, tolerability and human health effects of daily consumption	2 cups of *B. vulgaris* or *B. oleracea* consumed daily for 2 weeks by 26 healthy adults, randomly separated into 2 groups. 2 weeks wash-out period	Gastrointestinal tolerability: daily consumption is tolerable, no changes in GMB composition and abundance	[50]
	SFN from broccoli	Understand SFN metabolism and excretion	Administration of 100 μmol of SFN from fresh broccoli sprouts to 55 healthy people	Microbiome composition: positive correlation of SFN metabolites with bacteria of *Roseburia*, *Bifidobacterium, Bacteroides vulgatus* and *Ruminococcus torques*, and *Dorea longicatena* genera; negative correlation of SFN metabolites with *Alistipes* and *Blautia* genera	[48]
In vitro	SFN from broccoli (*Brassica oleracea* var. italica) seeds	Effects of SFN on gut microbiota composition and production of short-chain fatty acids	Simulated gastrointestinal digestion; in vitro fermentation (using fresh human faeces from obese, antibiotic-free volunteers) for 6, 12, and 24 h	Microbiome composition: Higher after 24 h in SFN-treated group compared to the SFN-free; higher abundance of *Firmmicutes*, *Weissella*, *Leuconostoc*, *Lactobacillus*, *Algiphilus* and *Faecalibacterium*; reduction of *Proteobacteria*, *Escherichia-Shigella*, *Klebsiella*, *Clostridium_sensu_stricto_1*, *Sutterella, Megamonas*, and *Proteus* in SFN-treated group	[49]
	SNG, GTP, GNT and their corresponding DS-GSLs (synthetic GSLs)	GSLs and their DS-GSLs metabolic fate after in vitro processing with *Lactobacillus agilis* R16, *Escherichia coli* VL8, and *Escherichia casseliflavus* CP1	24 h incubation with the compounds. GS-MS analysis	Products obtained from GSLs consumption: From SNG = AITC and ANIT (all 3 bacteria); From GTP = BZITC and BNIT (all 3 bacteria); From GNT = PEITC (all 3 bacteria) and PNIT (except *L. agilis*) Products obtained from DS-GSLs consumption: *L. agilis:* ANIT from DS-SNG; BNIT from DS-GNT; *E. casseliflavus* and *E.coli:* ANIT form DS-SNG; BNIT from DS-GTP; sPNIT form DS-GNT	[51]
	GTP (synthetic GSL)	Identification of activation of GSLs by *Bacteroides thetaiotaomicron* (*Bt*)	*Bt* strains VPI-5482, 8736, 7330, and 3731 cultured in a medium containing GTP. LC-MS/MS analysis	Identification of operon BT2159-BT2156 for GSLs conversion. Genes BT2158 and either BT2156 or BT2157 are required to get ITCs formation	[18]
	AITC, BZITC from the green parts of *Brassica* *carinata* and *Sinapis alba*	Study the formation of AITC and BZITC after the digestive process; effects of colonic fermentation on the formation of ITCs and their consequent impact on intestinal population	5 g of fresh samples or 2 g of lyophilised samples analysed after simulated gastrointestinal digestion and in vitro colonic fermentation (using faecal samples of three healthy donors); DNA extraction and sequencing	Final AITC and BZITC concentration after digestion and colonic fermentation: AITC reduction to 0.01 from fresh samples; 0.02 mg/g for lyophilised samples BZITC reduction to 0.1 Microbiome composition: Increase of *Bifidobacterium*, *Faecalibacterium*, *Blautia*, *Ruminocaccus*; slight increase of *Lactobacillaceae* in *Sinapis alba*; reduction of *Enterobacter* and *Klepsia*	[52]

Abbreviations: AITC: allyl isothiocyanate; ANIT: allyl nitrile; BZITC: benzyl isothiocyanate; BNIT: benzyl nitrile; DS-GSLs: desulfo-glucosinolates; GMB: gut microbiota; GNT: gluconarstutiin; GSL: glucosinolate; GTP: glucotropaeolin; ITCs: isothiocyanates; PEITC: Phenethyl-ITC; PNIT: phenylethyl nitrile; SFN: sulforaphane; SNG: sinigrin.

Due to the lack of in vivo studies, several in vitro investigations have been conducted and may help guide future research. In a study conducted by Sun et al. [49], SFN was isolated from broccoli and underwent in vitro simulated gastrointestinal digestion. After analysing the effects of simulated digestion on SFN stability, assessing that no changes occurred during these processes, they observed the effects of in vitro fermentation. After 6 h, the amount of SFN decreased significantly, remaining stable after 12 h. After 24 h, they analysed the bacterial community, evidencing that it was significantly higher in the group treated with SFN compared to the group without treatment, with an amelioration in the composition of the GMB: there was an increase in *Firmicutes*, the most abundant beneficial bacteria, and a reduction in *Proteobacteria*, which are indicators of intestinal imbalance [49]. An important study on GSL metabolism by human GMB was conducted by Luang-In et al. [51]. They evaluated the effects of *Enterococcus casseliflavus CP1*, *Lactobacillus agilis R16*, and *Escherichia coli VL8* on sinigrin, glucotropaeolin, gluconasturtiin, and their respective desulphated compounds. They observed that these bacteria were capable of degrading all the compounds, although at varying rates. The same GSLs incubated in culture media without bacteria did not undergo hydrolysis, confirming the role of bacterial activity in their metabolism. Degradation products were composed of ITCs and nitriles for what concerns GSLs and of nitriles alone for what concerns desulpho-GSLs. Liou et al. [18] investigated the method that *Bacteroides thetaiotaomicron* bacteria used to convert GSLs into ITCs. They identified the BT2159-BT2156 operon as essential for GSL metabolism, with the genes BT2157, BT2158, and BT2159 specifically involved in the formation of ITCs. They also observed that ITC formation does not inhibit the growth of *Bacteroides thetaiotaomicron*, as it happens with other bacteria like *Escherichia coli*. To confirm their hypothesis on the role of these genes in GSL metabolism, they inserted BT2157 and BT2158 genes in non-metabolising bacteria, such as *Bacteroides fragilis*. Results indicated that BT2157 and BT2158 alone are not able to convert GSLs into ITCs, but their expression in combination with either BT2156 or BT2159 led to the acquisition of GSL-metabolising capability. At the end, they underlined that in vitro conversion of GSLs to ITCs requires BT2158 coupled with either BT2156 or BT2157 [18]. In a recent study conducted by Cámara-Martos et al. [52], they evaluated the effect of GMB on the bioaccessibility of AITC and benzyl-ITC derived from white mustard (*Sinapis alba*) and Ethiopian mustard (*Brassica carinata*). The samples were divided into fresh and freeze-dried. After digestion, AITC from fresh samples had a final BF of 0.1 mg/g of DW, compared to an initial concentration of 0.1 mg/g, while benzyl-ITC had a BF of 0.3 mg/g of DW, compared to a total amount of 1.1 mg/g; lower values were obtained for both AITC and benzyl-ITC in freeze-dried samples. No increase was observed, either, after the addition of exogenous myrosinase. After that, they simulated colonic fermentation on the non-BF of *Brassica carinata* (the solid residue after simulated digestion) using both fresh and freeze-dried samples, obtaining a reduction of AITC levels to 0.01 and 0.02 mg/g, respectively. Similar results were obtained on the non-BFs of benzyl-ITCs. They suspected that the reduced concentration in lyophilised samples is lower due to a reduction in GSL concentration, rather than due to a lack of enzymatic activity, as demonstrated by the addition of myrosinase, which did not influence the results; on the contrary, the reduction in ITCs after colonic fermentation can be due to the activity of the intestinal flora. Moreover, the bacterial population appeared to be affected, with reported increases in the concentration of *Bifidobacteria* and decreases in *Enterobacteria* and *Klebsiella*. However, these observations are associative, and further research is needed to establish any causal relationships [52].

### 3.3. Food Matrix

Another important factor that affects the bioaccessibility and bioavailability of GSLs or ITCs is the food matrix. Unfortunately, only a few studies have been conducted to understand the importance not only of plant composition but also of the format in which the food is consumed and the ingredients or meals with which it is combined. A greater number of articles, however, have focused on how food pre-treatment can affect the final content. In a randomised crossover clinical trial [53], the impact of supplementing cooked broccoli with brown mustard seed powder (MSP) to increase the bioavailability of SFN has been explored. To overcome the limitation of myrosinase enzyme degradation by cooking, the authors designed a controlled human intervention study to evaluate whether the addition of an exogenous source of myrosinase could potentially restore or even increase SFN production in vivo. The trial recruited 12 healthy adult volunteers who consumed 200 g of cooked broccoli in two different conditions: with and without the addition of 1 g of brown mustard powder. The study followed a crossover design with a 1-week washout period between interventions to avoid the carryover effects. After consumption, 24 h urine samples were collected to quantify the bioavailability of SFN, measuring sulforaphane-N-acetyl-L-cysteine (SF-NAC), a stable urinary metabolite of SFN. The results showed that the mean urinary excretion of SF-NAC increased more than four times when mustard powder was added (44.7 ± 33.9 µmol/g creatinine) compared with 9.8 ± 5.1 µmol/g creatinine with broccoli alone (*p* < 0.05). The study showed a significant increase in SFN formation and bioavailability as well as elevated levels of its metabolite SF-NAC when myrosinase activity was exogenously provided. These enhancements are influenced by various factors, including plant processing methods, genetic variability, and individual differences in human metabolism, particularly glutathione S-transferase activity [53].

Oliviero et al. [54] tried to understand the effects of protein, lipids, and fibre gels on the bioavailability of ITCs, particularly SFN and iberin. They observed that lipids seem to facilitate ITCs absorption; moreover, when SFN and iberin were consumed with gels, their bioavailability was reduced compared to controls, with opposite results obtained when their precursors (GR and glucoiberin) were used. A similar study was conducted by Rungapamestry et al. [55], in which they evaluated different preparation methods of broccoli before consumption (microwaved for 2 min, microwaved for 5.5 min, or seed extract) with contemporary intake or non-intake of beef. Meals were given twice a week for three weeks to 12 healthy, non-smoking, human volunteers; urine samples were collected after each meal, just before the assumption of the following one. They were also given mustard sauce as a reference sample of AITC to check for inter-individual differences. Results showed that there were no significant differences between lightly cooked or fully cooked broccoli in terms of GSL composition, with a percentage of GR almost 20% of total GSL concentration (62.0 µmol/portion in lightly cooked broccoli compared to 71.7 µmol/portion in fully cooked broccoli). Myrosinase was less abundant in fully cooked broccoli (15.6 units/portion compared to 55.7 units/portion in lightly cooked broccoli). Both SFN and AITC were completely excreted within 24 h of consumption. They observed that, as what concerns AITC, the contemporary consumption of meat augmented its absorption, despite the fact that, in vitro, ITCs interact with proteins, letting the author hypothesise that a protein-rich meal would delay their absorption. The amount of SFN mercapturic acid was 5% of total GR intake for fully cooked broccoli and 20% of total GSL intake for lightly cooked broccoli, indicating that the latter gives a better bioavailability of the compound. In the case of broccoli seed extract (BSE), trace amounts of SFN mercapturic acid were detected in the excreted samples—at concentrations approximately 45 times higher than those present in the original extract. This observation is consistent with the findings of two similar studies on microencapsulation of Brassicaceae extracts that were performed by García-Ibañez et al. [56,57], who studied the microencapsulation, obtained using plant plasma membranes from cauliflower inflorescences (*Brassica oleracea* L. var. *botrytis*), of a red cabbage (*Brassica oleracea* L. var. *capitata f. rubra*), and of Bimi^®^ (combination of *Brassica oleracea* var. *italica* and *Brassica oleracea* var. *alboglabra* L., Morgan Hill, CA, USA) extracts to understand if this could ameliorate their bioaccessibility. To boost the GSL content of red cabbage, the plants were treated with methyl jasmonate (MeJA), effectively doubling the concentration of these compounds in the plant tissues employed for the extract. The nanoencapsulation step used plasma membrane vesicles to stabilise and protect the bioactive compounds, particularly SFN and indole-3-carbinol (I3C), during the dynamic in vitro gastrointestinal digestion model inoculated with the GMB of obese adults. For the Bimi^®^ extract, in vitro digestion was performed. The studies showed that the red cabbage nanoencapsulated extracts preserved higher levels of SFN and I3C over a 14-day digestion period than the unencapsulated extracts; the nanoencapsulated Bimi^®^ extract had a final SFN concentration six times higher compared to the non-encapsulated form after in vitro digestion. Moreover, the study on the human microbiota of patients treated with nanoencapsulated red cabbage extract had an unchanged overall composition, including the *Bacteroides/Firmicutes* ratio. A significant finding was that the increase in butyric acid production in the colon was significantly greater with the encapsulated extract. This short-chain fatty acid (SCFA) plays a crucial role in preserving gut health and has been associated with anti-inflammatory effects. The increased production of butyric acid may suggest that nanoencapsulated red cabbage extract might exert an effect on the butyrate-producing bacteria taxa in obese subjects. These findings showed that nanoencapsulation of red cabbage extract increases the stability and bioaccessibility of its bioactive compounds, leading to beneficial changes in the production of gut metabolites. Further in vivo investigations are required to validate the direct impact of the SCFA increase [56,57].

Zhu et al. [58] studied strategies to optimise the bioavailability of SFN from GR-rich BSE. A dynamic in vitro gastrointestinal digestion model and monolayers of Caco-2 cells, to simulate human intestinal absorption, were employed to evaluate the effects of the combination of BSE and myrosinase-rich MSP and ascorbic acid (AA), administered both as a free powder and in encapsulated formulations. The study showed that the acidic gastric environment inhibited myrosinase activity and limited the conversion of GR to SFN during gastric digestion. However, in the small intestine, the addition of exogenous myrosinase significantly increased the hydrolysis of GR to SFN. In particular, encapsulation of the BSE/MSP mixture (4:1 weight ratio) resulted in a 2.5-fold increase in the conversion efficiency compared with free powder administration, reaching a conversion rate of 72.1% compared with 29.3% for the free powder. Incorporating AA further improved the conversion efficiency and bioavailability of SFN, because AA acts as a cofactor and antioxidant, protecting myrosinase from oxidative degradation and maintaining its functional structure. In this study, adding AA to the BSE and MSP inhibited the formation of competitive hydrolysis products, such as nitriles and thiocyanates, which may occur without AA. This biochemical interaction explains why the addition of AA resulted in an increase in both the conversion efficiency of GR to SFN and the absorption of SFN through Caco-2 cells monolayers, supporting its crucial role in optimising the bioavailability of SFN in supplement formulations [58]. Recently, a study by Redha et al. [59] has uncovered the improvement in the bioaccessibility and bioavailability of SFN from broccoli through microencapsulation of two different protein matrices: whey protein isolate (BW) and pea protein isolate (BP). For this purpose, SFN was extracted from fresh broccoli and microencapsulated by freeze-drying with BW or BP as wall material. The study employed a dynamic in vitro gastrointestinal digestion model (SHIME) to assess the bioaccessibility of SFN, followed by a Caco-2/H29-MTX-E12 co-culture system to evaluate the intestinal bioavailability, transport, and absorption of SFN. The employment of this dual-model approach provided a physiologically relevant simulation of the human digestion and absorption process. The results showed that BW microencapsulation significantly improved SFN intake. The bioaccessibility of SFN from the BW formulation was 67.7%, compared with 19.0% from BP and 19.6% from dry broccoli powder. Concerning bioavailability, BW performed better, with 54.4% of SFN transported through the intestinal cell layer, compared with 9.6% of BP and 15.8% of dry broccoli. Furthermore, the apparent permeability coefficient (Papp) for SFN in the BW group was more than five times higher (1499 × 10-6 cm/s) than in BP and dry broccoli. This is underlying a significant improvement in intestinal absorption. These enhancements are probably due to beneficial interactions between SFN and specific amino acid residues in whey protein (e.g., valine, leucine, isoleucine), which provide stabilisation of the compound during digestion. Pea protein, mainly composed of globulins such as legumin and vicilin, showed less favourable encapsulation properties. In addition, whey protein may elicit peptides during digestion, further supporting its nutraceutical potential. Overall, this study suggests that WP is a promising encapsulation matrix for protecting and stabilising SFN during digestion. These results point to its potential application in the development of functional foods and nutraceuticals aimed at leveraging the chemopreventive and antioxidant properties of SFN. However, further research is needed to assess the stability of this formulation [59]. To conclude, a deeper understanding of GSL and ITC metabolism, bioavailability, and bioaccessibility sets the stage for examining how these mechanisms translate into measurable outcomes—an exploration undertaken in the following section through human intervention studies.

## 4. Intervention Studies on Glucosinolates and Isothiocyanates

### 4.1. Studies Based on Vegetable and Derivative Interventions

The potential health benefits associated with the consumption of Brassicaceae vegetables rich in GSLs, such as cabbage (*Brassica oleracea*), turnip (*Brassica rapa*), and Brussels sprouts (*Brassica oleracea* var. *gemmifera*), have attracted increasing interest. To investigate the effects of consuming cruciferous vegetables and their derivatives, most previous research is based on clinical and epidemiological studies using edible and commercially available parts of plants such as leaves, heads, or roots. However, despite the wide availability of literature on this topic, most clinical studies have focused mainly on broccoli, leaving the other Brassicaceae species relatively uninvestigated in human intervention studies in recent years. This lack of recent studies is largely due to the difficulty of standardising dosages, variability in preparation methods (e.g., raw or cooked), and the bioavailability of key compounds (GSLs) and their bioactive derivatives, ITCs. For example, a study carried out by Connolly et al. [60] highlighted that while preclinical evidence shows promising anti-inflammatory and antioxidant properties of GSLs, clinical trials in humans are still insufficient, due to the methodological heterogeneity and factors affecting the production of the bioactive compounds (ITCs), such as enzyme inactivation during cooking processes (Table 3).

**Table 3:** Dietary interventions including the intake of cruciferous vegetables and their derivatives.

Dietary Source	Sulphur-Nitrogen-Based Compounds	Biological Samples	Subject Characteristics	Study Record Identifier (NCT Number)	Metabolites and Conjugates–Concentration Range	Main Findings	Analytical Technique	Reference
*Cruciferous* vegetables	GSL, ITCs (e.g., SFN)	Blood, urine, tissue (preclinical and human)	Mixed: human (adults 30–60 yrs, both sexes, BMI 25–30 kg/m^2^).	N.A.	SFN metabolites: SFN-GSH, SFN-Cys, SFN-NAC, SFN-SULF. Plasma levels up to 0.5–2.0 µM; urinary excretion up to 70–80% of dose within 24 h.	Evidence of anti-inflammatory and anti-carcinogenic activity in preclinical models; some human trials support role in chronic disease prevention	HPLC, mass spectrometry, biomarker assays	[60]
Cruciferous vegetables	GR and SFN	Blood and urine	67 adults with mildly elevated BP (randomised crossover design)	ACTRN12618001010246 (VESSEL study)	SFN metabolites: SFN and metabolites: SFN-Cys, SFN-NAC, SFN-GSH, SFN-SULF quantified in plasma and urine	Significant BP reduction compared to control; improved vascular function markers	LC-MS/MS, BP monitoring	[61]
Bitter brassica vegetables (wild/traditional cultivars)	GSLs (higher levels due to cultivars)	Blood and urine	Type 2 diabetic patients, randomised control trial	N.A.	Not quantified	Improved glycemic control, insulin sensitivity, reduced inflammation markers	Clinical biochemistry assays, HOMA-IR	[62]

Abbreviations: GR: glucoraphanin; N.A.: not available; SFN: sulforaphane; I3C: indole-3-carbinol; SFN-GSH: sulforaphane–glutathione; SFN-Cys: sulforaphane–cysteine; SFN-NAC: sulforaphane–N-acetylcysteine; SFN-SULF: sulforaphane–sulfate; HPLC: high-performance liquid chromatography; LC-MS/MS: liquid chromatography–tandem mass spectrometry; ELISA: enzyme-linked immunosorbent assay; PCR: polymerase chain reaction; HOMA-IR: homeostasis pattern assessment of insulin resistance; BP: blood pressure; DSS: sodium dextran sulfate; TNBS: 2,4,6-trinitrobenzenesulfonic acid.

More recently, Alaba et al. [63] also pointed out that the small number of well-designed randomised controlled trials and the heterogeneity of study design complicated the possibility of drawing robust conclusions. Consequently, recent research has increasingly been based on preclinical models (in vitro and in vivo animal studies) to investigate the mechanistic pathways, and epidemiological studies investigating population-level correlations between cruciferous vegetable intake and the risk of occurrence of metabolic diseases (Table 3). Among the few recent clinical trials evaluating the impact of Brassicaceae vegetable consumption on cardiovascular health, a randomised controlled intervention study (VESSEL study), carried out by Connolly et al. [61], analysed the short-term effects of cruciferous vegetable intake on blood pressure in adults with slightly elevated systolic blood pressure (SBP; 120–160 mmHg) on eighteen participants with a mean age of 68 years. The study assessed the effects of a short-term diet high in cruciferous vegetables, which are rich in GSLs, including Brussels sprouts (*Brassica oleracea* var. *gemmifera*), cabbage (*Brassica oleracea* var. *capitata*), and kale (*Brassica oleracea* var. *sabellica*), as well as a control diet based on root and squash vegetables (e.g., potato, carrot, and pumpkin). Over two 2-week phases separated by a washout period, participants consumed 300 g/day of assigned vegetables as lunch and dinner meals, with adherence monitored through dietary logs and validated biomarkers such as S-methylcysteine sulfoxide (SMCSO) for cruciferous intake and plasma carotenoids for control. The GSL-rich intervention showed statistically significant reduction in 24 h SBP (2.5 mmHg, *p* = 0.002), mainly driven by a strong effect during daytime hours (3.6 mmHg, *p* < 0.001). Moreover, a moderate but significant improvement in serum triglyceride levels was observed (*p* = 0.04), suggesting a dual potential benefit on vascular and metabolic parameters. Another relevant clinical study was conducted in a parallel, randomised, controlled 12-week intervention involving 92 patients with type 2 diabetes to evaluate the differential effects of traditional and modern cultivars of Brassicaceae and root vegetables on metabolic health [62] (Table 3). Participants were randomly assigned to one of three groups. The first group consumed 500 g/day of bitter and strong-tasting (BST) vegetables, which included traditional cabbage cultivars such as white and red cabbage—vegetables particularly rich in GSLs—and root vegetables such as carrots, which provided additionally dietary fibre and micronutrient profile. The second group was given an equal daily amount (500 g/day) of mild and sweet-tasting (MST) cultivars, which were characterised by lower GSL content. Finally, the control group maintained the usual diet but received a small additional portion of 120 g/day of MST vegetables to maintain a minimal intake of plant foods without substantially altering dietary patterns. All vegetables were steamed to make them palatable and to preserve the phytochemical composition. Pre- and post-intervention assessments included fasting blood glucose (FBG) samples, oral glucose tolerance tests (OGTTs), 24 h blood pressure monitoring, and dual-energy X-ray absorptiometry (DEXA) scans. These results showed that both high-vegetable diets led to significant reductions in body mass index (BMI) and glycated haemoglobin levels compared with the control group (*p* < 0.05), indicating greater insulin sensitivity. Moreover, the BST group reported significant reductions in SBP and diastolic blood pressure (*p* < 0.05) compared with the MST and control groups. These effects are likely ascribed to the GSLs naturally found in BST vegetables that, once cut, chewed, and ingested, are enzymatically converted to ITCs, which are known for their antidiabetic, anti-inflammatory, and insulin-responsive activities [62].

### 4.2. Studies Based on Sprout Interventions

Cruciferous sprouts, particularly broccoli sprouts, have gained recognition as functional foods due to their naturally high content of GSLs and their potent bioactive derivatives, such as SFN. Their increasing popularity in the field of personalised nutrition is linked to their nutritional richness, low calorie content, and significant phytochemical profile [25].

In a randomised, double-blind, placebo-controlled clinical trial, participants were supplemented with two capsules daily of broccoli sprout powder (BSP, 200 mg/ capsule) or a placebo (maltodextrin) [64] (Table 4). The intervention lasted 12 weeks and included 288 participants, divided into three groups: Group A (hyperglycaemic individuals), Group B (overweight or obese individuals), and Group C (individuals with dyslipidaemia). The outcomes varied across the groups. In Group A, a reduction in postprandial blood glucose (PPBG) and low-density lipoprotein (LDL) cholesterol was observed, along with an improvement in the Physical Component Summary (PCS) score. In Group B, supplementation led to reductions in FBG, triglycerides, LDL cholesterol, and BMI, accompanied by an increase in high-density lipoprotein (HDL) cholesterol. Group C participants experienced significant reductions in BMI, triglycerides, LDL, and very low-density lipoprotein (VLDL) cholesterol. These findings suggest that BSP consumption positively modulates several markers associated with metabolic syndrome. The effects are likely mediated through its antioxidant and anti-inflammatory properties, attributed to the upregulation of phase II detoxification enzymes and activation of the Nrf2 signalling pathway [65,66]. Thus, this study supports the potential role of BSP as a functional dietary ingredient for the management of metabolic health risk factors [64]. Another double-blind, placebo-controlled clinical trial was conducted to investigate the potential anti-diabetic effects of BSE, which is rich in SFN, a compound known to modulate glucose metabolism [67]. Seventy-four drug-naive participants with prediabetes were randomised into two groups—BSE treatment and placebo—and received a daily oral dose (150 µmol of SFN) for 12 weeks. The primary outcome was a reduction in FBG levels. The trial demonstrated a statistically significant mean reduction of 0.2 mmol/L in FBG in the BSE group compared to placebo (*p* = 0.04), suggesting a modest hypoglycaemic effect. However, this outcome did not reach the predefined clinical threshold of a 0.3 mmol/L reduction. Exploratory subgroup analyses revealed that individuals with mild obesity, low insulin resistance, and impaired insulin secretion exhibited a greater therapeutic response, with an average FBG reduction of 0.4 mmol/L. This subgroup was classified as “responders”, indicating a possible phenotype that may benefit more substantially from BSE supplementation. In parallel, metagenomic sequencing of faecal samples was performed to characterise the GMB. Results indicated a distinct microbial profile in responders compared to non-responders. Notably, responders had a higher relative abundance of *Bacteroides* species, which encode a transcriptional regulator necessary for the enzymatic conversion of GR into bioactive SFN. This microbial feature was positively correlated with higher circulating SFN levels, suggesting that the efficacy of BSE may depend on the microbiota’s ability to bioactivate the compound. These findings highlight a host–microbiome interaction in modulating the metabolic response to BSE, where both the individual’s metabolic phenotype and microbial enzymatic capacity play crucial roles. Although the overall clinical effect of BSE on FBG reduction was limited, the identification of a responsive subgroup opens new avenues for personalised nutrition or microbiome-informed therapeutic strategies in prediabetes patients [67].

**Table 4:** Dietary interventions including the intake of cruciferous sprouts.

Dietary Source	Sulphur-Nitrogen-Based Compounds	Biological Samples	Subject Characteristics	Study Record Identifier (NCT Number)	Metabolites and Conjugates–Concentration Range	Main Findings	Analytical Technique	Reference
Broccoli sprout powder (200 mg per capsule, 2 capsules daily during 12 weeks)	GR	Blood (plasma/serum)	288 adults (18–60 years) in India with at least one metabolic risk factor	CTRI/2016/05/006977 (Indian Clinical Trials Registry)	N.A.	Significant reductions in fasting glucose, glycated haemoglobin, and triglycerides in the broccoli sprout group compared to placebo; improved metabolic profiles observed over 90 days	Standard clinical biochemistry assays for glucose, lipids, glycated haemoglobin	[64]
Broccoli sprout extract (12 weeks)	SFN ~150 µmol daily	Fasting blood; stool for gut microbiota	74 drug-naive adults (35–75 yr) with prediabetes, overweight/obese	NCT03763240	Serum SFN measured in subset; concentration correlated with gut BT2160 gene abundance	Modest glucose reduction overall (−0.2 mmol/L); greater effect (−0.4 mmol/L) in responders with mild obesity and low insulin resistance. Gut microbiota profile predicted response and was linked to higher SFN levels.	Standard biochemistry for fasting glucose and clinical biomarkers; microbiome sequencing; targeted assay for sulforaphane in blood	[67]
2 bottles of sprout juice per day (75 g of sprouts)	GR	Skeletal muscle biopsies; blood (plasma/serum)	9 healthy adults in combination with daily intense exercise	Not registered	N.A.	Broccoli sprouts combined with training lowered muscle oxidation and blood myeloperoxidase, reduced lactate in exercise, lessened night-time hypoglycaemia, and enhanced performance versus placebo	• Protein carbonylation in muscle: ELISA or spectrophotometric detection • Blood myeloperoxidase: immunoassay • Lactate during sub-maximal exercise: enzymatic assay • Continuous glucose monitoring	[68]
Broccoli sprout extract (200 µmol daily)	SFN 100 µmol/capsule	Blood (plasma/urine); prostate tissue biopsies	98 men scheduled for prostate biopsy	NCT01265953	SFN-GSH: 0.03/0.0002 µM (plasma/urine) SFN-CysGly: 0.04/0.005 µM SFN-Cys: 0.02/1.2 µM SFN-NAC: 0.03/2.9 µM	BSE supplementation increased SFN metabolites but did not significantly affect HDAC activity or prostate tissue biomarkers	QTRAP LC-MS/MS for SFN metabolites; RNA-Seq for gene expression; immunohistochemistry for tissue biomarkers	[69]
Broccoli sprout extract	ITC 200 µmol/day	Plasma, urine, breast tissue biopsies	30 postmenopausal women with stage I–II breast cancer	NCT01753908	Urinary ITC metabolites in BSE group: Pre-intervention: 15.9 ± 28.3 µmol/g creatinine Post-intervention: 228.0 ± 152.0 µmol/g creatinine Net change range: 6.6 to 598.7 µmol/g creatinine	BSE raised SFN metabolites and showed trends for increased tumour cell death markers and immune infiltration, with reduced proliferation markers	LC-MS/MS for SFN metabolites; immunohistochemistry for Ki-67, cleaved caspase-3, ER-α; proteomic analysis of morning urine	[70]
Broccoli sprout (15 caps/day)	Caps content: 90 mg SFN + 180 mg GR	Urine	40 patients with advanced, surgically non-resectable pancreatic ductal adenocarcinoma receiving palliative chemotherapy	NCT01879878 (POUDER pilot study)	N.A.	Feasibility confirmed despite high dropout and side effects; survival trend favoured treatment but not statistically significant	N.A.	[71]
White cabbage or pak choi sprouts beverage	1-cyano-2,3-epithiopropane (CETP), 1-cyano-3,4-epithiobutane (CETB), 1-cyano-4,5-epithiopentane (CETPent)	Blood and urine	9 (7 women and 2 men) Healthy adults aged 18−70 years with a BMI between 18 and 30 kg m^−2^	Not registered	CETP metabolite reached peak blood concentrations after 3 h of 70 nmol/L after cabbage and 12 nmol/L after pak choi sprout consumption.	CETP and CETPent rapidly absorbed and metabolised via the mercapturic acid pathway; CETB metabolite undetectable	UHPLC-TOF-MS	[72]

Abbreviations: GR: glucoraphanin; N.A.: not available; SFN: sulforaphane; SFN-GSH: sulforaphane–glutathione; SFN-CysGly: sulforaphane–cysteinyl–glycine; SFN-Cys: sulforaphane–cysteine; SFN-NAC: sulforaphane–N-acetylcysteine; LC-MS/MS, liquid chromatography coupled to mass spectrometry; HDAC: histone deacetylase; QTRAP LC-MS/MS quadrupole linear ion trap–liquid chromatography–tandem mass spectrometry; ITC: isothiocyanate; BSE: broccoli sprout extract; LC-MS/MS: liquid chromatography coupled to mass spectrometry; BMI: body mass index; CETP: 1-cyano-2,3-epithiopropane; CETPent: 1-cyano-4,5-epithiopentane; CETB: 1-cyano-3,4-epithiobutane; UHPLC-TOF-MS: ultra-high performance liquid chromatography–time-of-flight mass spectrometry; NCT: number or record study number assigned in ClinicalTrials.gov.

Another study investigated the effects of consuming GSL-rich broccoli sprout (GRS) supplements on oxidative stress and physiological adaptations to intense exercise training [68] (Table 4). In a randomised, double-blind, crossover design, nine healthy participants consumed either a GRS supplement (75 g of sprouts) or a placebo twice daily over a 7-day high-intensity interval training period. The findings revealed that GRS supplementation significantly reduced markers of oxidative stress, including carbonylated proteins in skeletal muscle and plasma myeloperoxidase levels, compared to the placebo condition. Furthermore, GRS intake led to reduced lactate accumulation during submaximal exercise and enhanced exercise performance, as indicated by a longer time to exhaustion during maximal exercise tests. At the molecular level, supplementation with GRS was associated with elevated Nrf2 protein levels in muscle tissue, suggesting activation of endogenous antioxidant defence mechanisms. In addition, GRS intake mitigated nocturnal hypoglycaemic episodes and lowered average blood glucose levels, indicating improved glucose regulation during intense training. Collectively, these results suggest that GRS supplementation may enhance physiological adaptations to high-intensity exercise by reducing oxidative stress and supporting metabolic homeostasis [68].

A study carried out by Zhang et al. [69] investigated the effects of BSE, rich in SFN, on prostate cancer-related biomarkers in men undergoing prostate biopsy (Table 4). In this randomised, double-blind trial involving 98 participants, those receiving 200 µmol of BSE daily showed significantly higher levels of SFN metabolites in their urine and plasma compared to the placebo group, indicating good bioavailability. Gene expression analysis of prostate tissue revealed that BSE intake was associated with changes in the expression of 40 genes, including downregulation of α-methylacyl-CoA-racemase (AMACR) and androgen receptor-regulated long noncoding RNA 1 (ARLNC1), which are linked to prostate cancer. However, no significant changes were observed in HDAC activity or in key prostate cancer biomarkers such as Ki-67 or p21. Interestingly, among participants with confirmed prostate cancer, BSE supplementation was associated with increased HDAC activity. Only a few participants showed detectable SFN in prostate tissue, suggesting limited local accumulation. The treatment was safe and well tolerated. Overall, while BSE influenced systemic SFN levels and gene expression, it did not significantly affect HDAC activity or most prostate cancer biomarkers [69]. Other studies have also explored the effects of broccoli sprouts on cancers beyond prostate cancer, such as the randomised, placebo-controlled trial conducted by Wang et al. [70]. This study involved 30 postmenopausal women with breast cancer who received either 200 µmol/day of BSE or a placebo for two weeks before surgery. Tissue analysis in the BSE group showed trends toward increased levels of cleaved caspase-3 and tumour-infiltrating lymphocytes (TILs), along with reduced Ki-67 expression and a lower nuclear-to-cytoplasmic ratio of oestrogen receptor alpha (ER-α). These changes suggest enhanced apoptosis, an augmented immune response, and decreased cell proliferation and ER-α-mediated signalling. Urinary proteomic analysis identified over 100 BSE-specific protein alterations, implicating 55 enriched signalling pathways, including the Nrf2-mediated oxidative stress response and acute phase response pathways. Network analysis predicted upstream regulators such as serum response factor (SRF), IL-6, and myocardin-related transcription factor B (MRTFB), which may contribute to the observed effects on apoptosis and immune activation [70].

A pilot study (POUDER) investigated the potential effects of broccoli sprout supplementation in patients with advanced pancreatic ductal adenocarcinoma (PDA) undergoing palliative chemotherapy [71] (Table 4). The study included 40 participants, with 29 assigned to the treatment group and 11 to the control group. Patients in the treatment group received 15 capsules daily, each containing 90 mg of SFN and 180 mg of GR, while the placebo group received methylcellulose capsules. The study aimed to assess overall survival, patient-reported outcomes, and the feasibility of long-term supplementation. The results showed a higher dropout rate in the treatment group (72%) compared to the placebo group (55%). The most commonly reported reasons for discontinuation were gastrointestinal symptoms, including nausea, vomiting, and flatulence, which some participants attributed to the broccoli sprout capsules. Despite these issues, the treatment group exhibited a lower mortality rate within the first 180 days (25%) compared to the placebo group (43%). Kaplan–Meier analysis suggested a survival benefit in the treatment group during this period, although the difference was not statistically significant (*p* = 0.3). While the findings indicate that supplementation may offer potential survival benefits, the high dropout rate and reported side effects highlight the need for further research to improve tolerability and adherence in this patient population [71]. Recent findings have provided insights into the metabolism, bioavailability, and urinary excretion of epithionitriles—specifically 1-cyano-2,3-epithiopropane (CETP), 1-cyano-3,4-epithiobutane (CETB), and 1-cyano-4,5-epithiopentane (CETPent) [72]. In a controlled clinical study, nine healthy participants consumed beverages made from white cabbage sprouts or pak choi, each containing one of the three epithionitriles on separate occasions. The study revealed that CETP and CETPent were rapidly absorbed and primarily metabolised via the mercapturic acid pathway, with urinary recovery rates of approximately 28 ± 9% for CETP and 12 ± 3% for CETPent. These compounds were identified in urine as N-acetylcysteine (NAC) conjugates, indicating efficient biotransformation and clearance. In contrast, no urinary metabolites of CETB were detected, suggesting poor absorption, rapid degradation, or metabolism through an as-yet unidentified pathway. Plasma concentrations of all compounds remained low, supporting the notion of rapid metabolism and elimination. These findings demonstrate, for the first time in humans, that certain GSL-derived epithionitriles are bioavailable and actively metabolised, while others may follow alternative metabolic routes. This study enhances our understanding of the complexity of GSL-derived compound metabolism in humans [72].

### 4.3. Studies Based on Enriched Food and Extract Interventions

Some recent intervention studies significantly highlight the advances in understanding the bioavailability and metabolism of GSLs and ITCs using enriched foods and standardised extracts (Table 5). These studies aim to provide controlled and quantified intakes of these bioactive compounds, allowing a more precise investigation of dose–response and cross-individual variability. Recent research has pointed out that the food matrix may enhance or hinder the bioavailability of these compounds as previously stated [37,73,74]. A randomised trial evaluated the impact of supplementation of a GR-rich broccoli sprout extract on liver function in healthy middle-aged adults with normal hepatic biomarkers. A total of 98 participants aged 45–65 years were enrolled and randomly assigned to receive a daily dose of 60 mg of GR or a placebo over a 24-week intervention period [75]. The main goal of the study was to assess whether taking the supplement could reduce blood levels of alanine aminotransferase (ALT), an enzyme commonly used to indicate liver function; high ALT levels often suggest stress or liver damage. The primary endpoint was the change in serum ALT levels over a 24-week period, which showed a statistically significant decrease (*p* = 0.04) in the intervention group, compared with the placebo, suggesting an enhancement in liver function likely mediated by SFN. The secondary endpoints involved serum ALT at early time points (4–12 weeks), serum aspartate aminotransferase (AST) and γ-glutamyl transferase (γ-GTP) after 4–12 weeks, and serum GSH levels after 4–12 weeks and 24 weeks. While γ-GTP levels showed a decreasing trend in the treated group, changes in AST and GSH did not reach statistical significance between both groups. Importantly, the supplement was well tolerated, and no serious adverse effects were reported. These findings suggest that regular intake of GR-enriched broccoli sprout extract has the potential to confer hepatoprotective effects in subjects at risk of developing liver dysfunction, potentially through the activation of cytoprotective pathways such as Nrf2. However, the modest effect size and lack of significant changes in several secondary biomarkers suggest that additional studies are needed to clarify the long-term clinical relevance and underlying mechanisms.

**Table 5:** Dietary interventions including the intake of enriched foods and extracts.

Dietary Source	Sulphur-Nitrogen-Based Compounds	Biological Samples	Subject Characteristics	Study Record Identifier (NCT Number)	Metabolites and Conjugates–Concentration Range	Main Findings	Analytical Technique	Reference
Broccoli sprout supplements	GR	Blood	Healthy middle-aged adults, high-normal liver markers	N.A.	No direct measurement of circulating metabolites; significant reduction in γ-GTP compared to control, with favourable trends in ALT and AST	Improved liver enzyme levels post-intervention	Clinical blood chemistry analysis	[75]
Freeze-dried cabbage bar	GSLs	Blood	T2D patients, double-blinded RCT	N.A.	No direct measurement of GSLs or metabolite concentrations in blood; clinical outcomes include reduced HbA_1_c, body weight, and calorie intake	Improved glucose control and insulin response	Biochemical clinical assays	[76]
*Descurainia sophia* seed extract	GSLs (assumed)	Blood (thyroid-related markers)	Hyperthyroid patients, pilot RCT	N.A.	No direct measurement of GSLs or metabolite concentrations in blood. *D. sophia* extract results in increase TSH compared to placebo	Reduction in thyroid hormone levels (T3, T4)	Hormonal assays (ELISA, RIA)	[77]
SFN + Alliin (broccoli + garlic)	SFN, Alliin	Prostate tissue (biopsy)	Humans undergoing prostate biopsy	N.A.	Tissue concentration measured: Total ITC metabolites 1.1 nmol/g tissue. SFN: 0.4 nmol/g. SFN-NAC: 0.6 nmol/g	SFN and alliin detected in prostate tissue after diet	Tissue LC-MS/MS analysis	[78]
*Nasturtium officinale* extract	GSLs	Blood (oxidative/inflammatory markers)	People with physical disability, double-blind RCT	N.A.	No specific metabolite concentrations reported	Reduced oxidative stress and inflammation biomarkers	ELISA, biochemical markers	[79]
Cruciferous vegetables (general intake)	GSLs	Urine (2/16α-hydroxyestrone ratio)	Premenopausal women	N.A.	Urinary 2/16α-hydroxyestrone ratio measured; no significant changes observed after cruciferous vegetable intake	No significant effect on estrogen metabolite ratio	Urine LC-MS analysis	[80]
Maca extract (*Lepidium meyenii*)	Benzyl glucosinolate	Blood, fatigue questionnaires	Women, fatigue symptoms, RCT	N.A.	No specific metabolite concentrations reported	Significant reduction in fatigue with maca extract	Subjective fatigue scales + blood analysis	[81]
Supplement with GSLs + flavonoids	GSLs, phytosterols, flavonoids	Blood (safety, tolerance)	Adult women, Phase I RCT	N.A.	No specific metabolite concentrations reported	Safe and well tolerated across doses	Clinical safety and tolerability assays	[82]

Abbreviations: GR: glucoraphanin; GSLs: glucosinolates; SFN: sulforaphane; SFN-NAC: sulforaphane–N-acetylcysteine; SFN-GSH: sulforaphane–glutathione; SFN-Cys: sulforaphane–cysteine; SFN-CysGly: sulforaphane–cysteinyl–glycine; γ-GTP: gamma-glutamyl transpeptidase; ALT: alanine aminotransferase; AST: aspartate aminotransferase; HbA_1_c: glycated hemoglobin; TSH: thyroid-stimulating hormone; T3: triiodothyronine; T4: thyroxine; ITC: isothiocyanate; LC-MS/MS: liquid chromatography–tandem mass spectrometry; ELISA: enzyme-linked immunosorbent assay; RIA: radioimmunoassay; RCT: randomised controlled trial; N.A.: not available.

Another clinical intervention study that assesses the health effects of cruciferous vegetables is a randomised, double-blind, placebo-controlled trial which evaluated the health impact of freeze-dried cabbage (*Brassica oleracea* var *capitata*) on patients with type 2 diabetes [76]. The study recruited 30 adults who were diagnosed with type 2 diabetes and randomly assigned them to receive either a freeze-dried cabbage bar daily or a placebo bar for a total period of 12 weeks. The freeze-dried formulation aimed to preserve the bioactive compounds of vegetables while ensuring easy administration and homogeneity. Participants in the intervention group consumed bars of 26.3 g of freeze-dried cabbage powder daily (corresponding to about 341 g of fresh cabbage daily). Both groups continued their usual diet, ensuring that any changes in clinical parameters could be attributed to the intervention. The results showed that administration led to significant improvements in glycaemic control, as highlighted by a mean reduction in glycated haemoglobin of 1.4 ± 0.9 mmol/mol compared to an increased mean of 1.4 ± 0.8 mmol/mol in the control group (*p* = 0.04). Insulin resistance, as assessed by Homeostasis Model Assessment of Insulin Resistance (HOMA-IR), also improved significantly. The intervention group showed a reduction of 1.0 ± 0.3 compared with an increase of 0.2 ± 0.3 in the control group (*p* = 0.03). FBG decreased by 0.5 ± 0.8 mmol/L in the intervention group and increased by 0.9 ± 0.4 mmol/L in the control group (*p* = 0.2). Furthermore, LDL-C levels were relatively stable in the intervention group and slightly increased in the control group, showing no significant differences. Overall, these results suggest that regular consumption of cabbage in the daily diet may benefit glycaemic regulation in subjects with type 2 diabetes. In a pilot, randomised double-blind placebo-controlled clinical trial, Farzameh et al. [77] evaluated the additive effects of one 350 mg/daily *Descurainia sophia* seed extract capsule in 10 newly diagnosed hyperthyroid women receiving standard methimazole therapy. Over a 60-day treatment period, primary clinical and biochemical endpoints were evaluated. The *Descurainia sophia* group demonstrated a reduction in serum free thyroid hormone 3 (T3) (from 14.5 ± 9.1 × 10^−9^ to 9.1 ± 3.8 × 10^−9^ mol/L) and free T4 (from 31.8 ± 3.7 × 10^−9^ to 29.1 ± 3.1 × 10^−9^ mol/L), compared with the placebo cohort, which maintained persistently high levels. Thyroid-stimulating hormone (TSH) increased significantly in the treatment (from 0.05 ± 0.02 to 4.7 ± 0.1 mIU/L), reflecting a statistically significant reversal of thyrotoxic suppression (*p* < 0.05). Clinically, patients in the *Descurainia sophia* arm reported significant improvement in symptoms of thyrotoxicosis (*p* < 0.05). These results suggest that the GSL constituents of *Descurainia sophia* can effectively inhibit iodine absorption, thereby improving standard antithyroid therapy and promoting biochemical normalisation in hyperthyroid patients.

In another randomised, double-blind, factorial 2 × 2 intervention, Livingstone et al. evaluated tissue accumulation of SFN and alliin in 39 men undergoing trans-perineal prostate biopsy [78]. Participants were assigned to one of four supplement arms—GR (BroccoMax^®^, Jarrow Formulas, Chatsworth, CA, USA), alliin (Kwai Heartcare^®^, Bury St Edmunds, UK), both, or a placebo—for 4 weeks before biopsy. Transition and peripheral zones of prostate tissue were analysed for SFN and its thiol conjugates as well as alliin and metabolites. Subjects who received GR showed a highly significant increase in SFN in both zones (*p* < 0.0001), similar to levels previously observed in breast tissue after similar supplementation. This study provides evidence that SFN accumulates in human prostate tissue following dietary supplementation, suggesting a plausible localised in situ chemoprotective mechanism [78]. Clemente et al. [79] in a double-blind, placebo-controlled clinical trial evaluated the impact of a standardised extract of *Nasturtium officinale* (watercress; SENO) on oxidative stress and biomarkers of inflammation in 65 participants: 15 non-disabled controls (evaluated once) and 50 individuals with physical disabilities were randomised to receive SENO (750 mg/kg/day) or a placebo for 5 weeks [79]. Serum samples collected at baseline (day 0) and after surgery (day 36) were analysed for lipid peroxidation (TBARS), protein carbonyls, antioxidant enzymes (catalase, SOD), and C-reactive protein (CRP). Compared to the placebo group, the SENO-treated group showed significant reductions in several endpoints: TBARS decreased by 25%, protein carbonyls by 20%, and CRP levels by 15%. Enzymatic activities also shifted: catalase and SOD levels decreased, indicating a reduction in oxidative load, supporting the antioxidant effect of SENO [79]. However, the reduction in oxidative and inflammatory biomarkers provides preliminary evidence of the therapeutic potential of SENO in this population, which requires further large-scale validation studies.

In a placebo-controlled study, Davis et al. [80] examined whether cruciferous vegetable intake alters the urinary ratio of 2 to 16α-hydroxyestrone, a putative biomarker of breast cancer risk, in healthy premenopausal women aged 38 to 50 years with basal ratios ≤ 3.0. Seventy-eight participants were randomised to one of three arms: 6 capsules/day of dried cabbage and Brussels sprouts, or a placebo, for 8 weeks. Urinary 2:16 ratios were measured at baseline, after 4 weeks, and after 8 weeks and analysed using repeated measures ANOVA. Neither the main effect of treatment (*p* = 0.9) nor the treatment/time interaction (*p* = 0.6) were significant. However, a significant overall time effect (*p* = 0.02) was observed. Pearson correlation analyses also revealed that android fat distribution and android–gynoid fat ratio predicted changes in the urinary 2:16 ratio (*p* ≤ 0.05) [80]. Therefore, moderate intakes of cruciferous vegetables, either through supplements of fresh products, do not significantly modulate oestrogen metabolism, as reflected by the 2:16 urinary ratio, over two months in this population, and emphasise the importance of accounting for temporal variations and body composition in future study designs.

In a similar type of study (randomised, double-blind, placebo-controlled, parallel-group), Honma et al. [81] evaluated the anti-fatigue effect of a maca extract (*Lepidium meyenii*), standardised to contain 9.6 mg/day of benzyl-GSL in 60 adult women for a 4-week intervention. Subjects were randomly assigned to maca extract (400 mg/day, equivalent to 9.6 mg benzyl glucosinolate) or placebo, and anti-fatigue was assessed using a visual analogue scale (VAS) from 0 to 10 at baseline and at the end of the study. While both groups showed a reduction in fatigue scores, stratified analysis revealed that women under age 45 who received maca experienced a significantly greater decrease in VAS than the placebo group. The results suggest a statistically and clinically significant anti-fatigue response in younger women, consistent with preclinical findings in which benzyl glucosinolate improved endurance through increased fatty acid utilisation and glycogen preservation. Safety evaluation showed no adverse events or clinically relevant changes in blood parameters, suggesting that 400 mg/day of this extract is well tolerated. These preliminary results support benzyl-GSL-enriched maca as a promising candidate for reducing daily fatigue in young women, which deserves to be further explored in larger cohorts with objective performance metrics.

A parallel phase I study by Villar-López et al. [82] evaluated the safety and tolerability of a natural herbal supplement (Warmi^®^, Lima, Peru) containing GSLs, phytosterols, and citrus flavonoids in healthy women aged 18–40 years. A total of 55 participants were randomised to receive either 1.7 mg/day, 3.3 mg/day of supplement, or a matching placebo (3.3 mg/day) for 12 weeks, with monthly clinical evaluations and complete laboratory analysis. All participants completed the study with no reports of adverse events. Results revealed that the 1.7 mg/day dose was associated with modest increases in FBG (0.3 mmol/L) and uric acid (0.02 mmol/L), while the 3.3 mg/day group showed slight reductions in respiratory rate (2 breaths/min), increases in basal body temperature (0.2 °C) and a slight increase in SBP (4 mm Hg), all statistically significant compared to the placebo (*p* < 0.05) but considered clinically insignificant. Overall, the supplement demonstrated a beneficial safety and tolerability profile at doses up to 3.3 mg/day for 3 months, supporting its suitability for further efficacy studies.

## 5. Conclusions and Future Trends

In conclusion, the clinical evidence reviewed underscores the promising role of cruciferous vegetables as functional foods capable of delivering bioactive compounds derived from GSLs, such as SFN and epithionitriles. These phytochemicals have demonstrated anti-inflammatory, antioxidant, and chemo-preventive properties in various populations, including individuals with metabolic disorders and cancer. Despite these encouraging findings, many of the studies were conducted with relatively small sample sizes and short intervention durations, which limit both the generalisability and long-term applicability of the results. Moreover, considerable heterogeneity across trials—regarding study design, formulation types, exogenous myrosinase, dosing regimens, and outcome measures—complicates direct comparisons and limits the feasibility of robust meta-analyses [83].

The nature of the interventions, often involving whole foods or minimally processed products, further impedes the implementation of blinding and placebo controls, which are essential to minimise bias in clinical research. Additionally, significant interindividual variability—driven by factors such as dietary patterns, genetic polymorphisms, GMB composition, and metabolic phenotype—was frequently observed but not consistently accounted for in study designs. Another key limitation is the inconsistent application of biomarkers to assess bioavailability and biological response, which hampers the development of standardised criteria for evaluating efficacy [83]. Tolerability concerns, particularly gastrointestinal side effects reported in certain patient populations, also emphasise the need to refine formulation strategies and adopt patient-centred approaches to intervention design.

To fully realise the health-promoting potential of cruciferous vegetables and their derivatives within the framework of precision nutrition, large-scale, long-term clinical trials are needed. These studies should aim to validate health outcomes, optimise delivery systems, and elucidate the complex host–compound–microbiome interactions that drive individual variability in response. However, translating clinical findings into actionable public health recommendations remains a challenge, due to issues such as formulation standardisation, consistent dosing, and product stability and shelf-life constraints. These limitations collectively highlight the pressing need for more rigorous, harmonised methodologies in the design and implementation of clinical trials, in order to support the integration of cruciferous-derived interventions into evidence-based strategies for disease prevention and health promotion.

In this context, future studies should be designed with robust, randomised, placebo-controlled protocols that account not only for the standardisation of GSL- and ITC-derived formulations but also for the selection of appropriate biomarkers, stratification of participants based on genetic and microbiome profiles, and thorough assessment of tolerability. An efficient protocol for human studies would ideally include (i) a well-characterised and stable formulation with clearly defined active compounds and dosing; (ii) participant stratification according to relevant individual factors (e.g., diet, genotype, GMB); (iii) validated biomarkers of exposure and biological effect; and (iv) sufficient duration and follow-up to evaluate both efficacy and safety. Incorporating these elements will help bridge the gap between mechanistic research and real-world application, ultimately enhancing the translational impact of interventions based on GSL and ITCs.

## Figures and Tables

**Table 1:** The most abundant GSLs and their cognate isothiocyanate (ITC) derivatives. Extracted and modified from previous investigations [3,6,15,16].

Glucosinolate (GSL)	Molecular Structure	Isothiocyanate (ITC)	Molecular Structure	Cruciferous Vegetable
Glucobrassicin, Neoglucobrassicin	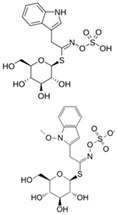	Indole-3-carbinol (I3C) 3,3-diindolylmethane (DIM)	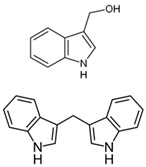	All crucifers
Sinigrin	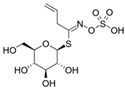	Allyl isothiocyanate (AITC)	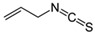	Mustard Brussels sprouts Cauliflower
Glucoraphanin	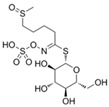	Sulforaphane (SFN)	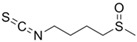	Broccoli Arugula
Gluconasturtiin	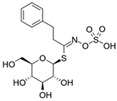	Phenyl isothiocyanate (PEITC)	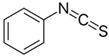	Cabbage Chinese cabbage Radish Watercress
Glucotropaeolin	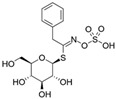	Benzyl isothiocyanate (BITC)	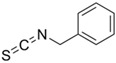	Garden cress Horseradish White mustards
Sinalbin, Glucosinalbin	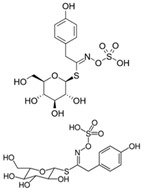	4-hydroxybenzyl isothiocyanate	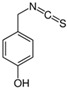	White mustards
Progoitrin Epiprogoitrin	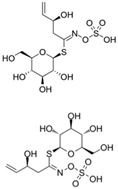	5-vinyloxazolidine-2-thione/Goitrin	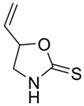	Brussels sprouts Salad rocket Sea kale Turnips
Glucoerucin	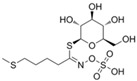	Erucin	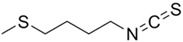	Salad rocket Radish Chinese cabbage Wild rocket

## Abbreviations

The following abbreviations are used in this manuscript:

AA	Ascorbic acid
AITC	Allyl-isothiocyanate
ALT	Alanine aminotransferase
AMACR	α-methylacyl-CoA-racemase
ARLNC1	Androgen receptor-regulated long noncoding RNA 1
AST	Aspartate aminotransferase
BF	Bioaccessible fraction
BMI	Body mass index
BP	Pea protein isolate
BSE	Broccoli seed extract
BSP	Broccoli sprout powder
BST	Bitter and strong-tasting vegetable
BW	Whey protein isolate
CETB	1-cyano-3,4-epithiobutane
CETP	1-cyano-2,3-epithiopropane
CETPent	1-cyano-4,5-epithiopentane
CRP	C-reactive protein
DEXA	dual-energy X-ray absorptiometry
DNMTs	DNA methyltransferases
DW	dry weight
ER-α	oestrogen receptor alpha
FBG	fasting blood glucose
γ-GTP	γ-glutamyl transferase
GSLs	Glucosinolates
GMB	Gut microbiota
GR	Glucoraphanin
GRS	Glucosinolate-rich broccoli sprout
GSH	Glutathione
HDAC	Histone deacetylase
HDL	High-density lipoprotein
HOMA-IR	Homeostasis Model Assessment of Insulin Resistance
I3C	Indole-3-carbinol
IL	Interleukin
Keap1	Kelch Like ECH Associated Protein 1
ITCs	Isothiocyanates
LDL	Low-density lipoprotein
MAPK	Mitogen-activated protein kinase
MeJA	Methyl jasmonate
MITC-1	Moringin
MRTFB	Myocardin-related transcription factor B
MSP	Mustard seed powder
MST	Mild and sweet-tasting vegetable
NAC	N-acetylcysteine
NF-κB	Nuclear factor kappa B
Nrf2	Nuclear factor erythroid 2-related factor 2
OGTT	Oral glucose tolerance test
Papp	Apparent permeability coefficient
PCS	Physical Component Summary
PEITC	Phenyl-ITC
PPBG	Postprandial blood glucose
SBP	Systolic blood pressure
SCFA	Short-chain fatty acid
SENO	Standardised extract of Nasturtium officinale
SF-NAC	Sulforaphane-N-acetyl-L-cysteine
SFN	Sulforaphane
SMCSO	S-methylcysteine sulfoxide
SRF	Serum response factor
TILs	Tumour-infiltrating lymphocytes
TNF-α	Tumour necrosis factor-α
TSH	Thyroid-stimulating hormone
VAS	Visual analogue scale
VLDL	Very low-density lipoprotein
